# Intraorbital pressure–volume characteristics in a piglet model: In vivo pilot study

**DOI:** 10.1371/journal.pone.0296780

**Published:** 2024-01-12

**Authors:** Yasin Hamarat, Laimonas Bartusis, Vilma Putnynaite, Rolandas Zakelis, Mantas Deimantavicius, Vilma Zigmantaite, Ramunė Grigaleviciute, Audrius Kucinskas, Evaldas Kalvaitis, Arminas Ragauskas

**Affiliations:** 1 Health Telematics Science Institute, Kaunas University of Technology, Kaunas, Lithuania; 2 Biological Research Center, Lithuanian University of Health Sciences, Kaunas, Lithuania; University of Florida, UNITED STATES

## Abstract

Intracranial pressure measurement is frequently used for diagnosis in neurocritical care but cannot always accurately predict neurological deterioration. Intracranial compliance plays a significant role in maintaining cerebral blood flow, cerebral perfusion pressure, and intracranial pressure. This study’s objective was to investigate the feasibility of transferring external pressure into the eye orbit in a large-animal model while maintaining a clinically acceptable pressure gradient between intraorbital and external pressures. The experimental system comprised a specifically designed pressure applicator that can be placed and tightly fastened onto the eye. A pressure chamber made from thin, elastic, non-allergenic film was attached to the lower part of the applicator and placed in contact with the eyelid and surrounding tissues of piglets’ eyeballs. External pressure was increased from 0 to 20 mmHg with steps of 1 mmHg, from 20 to 30 mmHg with steps of 2 mmHg, and from 30 to 50 mmHg with steps of 5 mmHg. An invasive pressure sensor was used to measure intraorbital pressure directly. An equation was derived from measured intraorbital and external pressures (intraorbital pressure = 0.82 × external pressure + 3.12) and demonstrated that external pressure can be linearly transferred to orbit tissues with a bias (systematic error) of 3.12 mmHg. This is close to the initial intraorbital pressure within the range of pressures tested. We determined the relationship between intraorbital compliance and externally applied pressure. Our findings indicate that intraorbital compliance can be controlled across a wide range of 1.55 to 0.15 ml/mmHg. We observed that external pressure transfer into the orbit can be achieved while maintaining a clinically acceptable pressure gradient between intraorbital and external pressures.

## Introduction

Intracranial pressure (ICP) is the pressure of fluids such as the cerebrospinal fluid (CSF) within the skull [1, 2]. The measurement of ICP is commonly used as a tool in neurocritical care to diagnose and monitor conditions such as traumatic brain injury (TBI), stroke, and intracranial tumors. Continuous ICP monitoring is crucial for certain groups of patients who could have sudden pathophysiological ICP variation, such as TBI patients [3]. While ICP monitoring is considered an important tool in the management of these conditions, it is not always predictive of structural and functional neurological deterioration [4]. ICP monitoring cannot directly measure or reflect the actual damage that the brain tissue may be experiencing, which can result in cases where patients who had normal ICP readings still suffering from too low intracranial compliance (ICC).

The Monro-Kellie doctrine is a fundamental concept in neurology that describes the relationship between ICP and the volume of brain tissue, cerebrospinal fluid (CSF), and blood in the intracranial compartment [5, 6]. The relationship between intracranial volume and pressure is governed by this doctrine [7, 8]. Therefore, the doctrine highlights the importance of maintaining normal intracranial pressure and the significance of understanding ICC and elastance.

ICC and intraorbital compliance (IORC) are two important physiological parameters that influence the pressure and volume changes in the brain and orbit, respectively [9]. ICC refers to the ratio of the change in intracranial volume to the change in ICP. IORC relates to the ratio of the change in intraorbital volume (IOV) to the change in intraorbital pressure (IORP). ICC plays a significant role in maintaining cerebral blood flow, and cerebral perfusion pressure [10].

ICC reflects the capacity of the skull contents to expand or contract in response to volume changes in cerebral blood, brain tissue, and cerebrospinal fluid. A decrease in ICC can lead to cerebral ischemia, herniation, and ultimately, irreversible neurological damage. Increased ICP can lead to a serious life-threatening medical condition such as worsening of intracranial pathology [4] and may be fatal. Thus, monitoring ICC in addition to ICP can aid clinicians in identifying patients who are at high risk of developing neurological complications and guide the decision-making process for treatment [11, 12].

We hypothesize the possibility of non-invasive measurement of intracranial compliance by identifying the equilibrium between ICC and IORC. However, this remains speculative given the absence of data on the relationship between ICC and IORC. The hypothesis could be achieved through the replication of intracranial pressure–volume dynamics within the intraorbital structure by incrementally introducing predetermined volumes into the pressure chamber. In this study, we tested the possibility to transfer external pressure into the eye orbit with a clinically acceptable gradient of intraorbital and external pressure in a large-animal model as a part of ongoing clinical studies. We also experimentally tested the possibility of managing intraorbital compliance and changing it in a wide range by applying an external pressure to the closed eyelid of an animal.

## Methods

### Preparation of animals

A local Lithuanian breed of piglets was used in this study. Animals were housed in an accredited animal-use facility at the LUHS Biological Centre in Kaunas, Lithuania. Piglets were obtained from a licensed supplier, adapted to the facility environment, and maintained for a minimum of 7 days. The study was approved by the national board for the use of laboratory animals (registration no. G2−186), and animal care complied with the European Commission Requirements for Use of Laboratory Animals. Piglets were anesthetized using 3 mg/kg of xylazine hydrochloride (Sedaxylan, Eurovet Animal Health, Bladel, Netherlands), 20 mg/kg of ketamine hydrochloride (Ketamidor, Richter Pharma, Wels, Austria) and 3 μg/kg of fentanyl citrate (Fentanyl Kalceks, AS Kalceks, Riga, Latvia).

Piglets were inducted intravenously using 8 mg/kg of sodium thiopental (Thiopental VUAB, VUAB pharma A.S., Roztoky, Czech Republic) and intubated using a 6-inch cuffed tracheal tube (Intersurgical Ltd, Wokingham, UK). During all procedures, analgesic was injected continuously using an infusion pump (Draeger, Lubeck, Germany), and general anaesthesia was maintained using inhaled sevoflurane (Sevoflo, Abbot, IL, USA). Electrocardiogram, arterial blood pressure, respiration, temperature, and oxygen saturation were monitored, and the stable condition of each animal was ensured. The ventilation volume and frequency were controlled at 8 ml/kg and 12 breaths/min, respectively, and body temperature was maintained with a thermal blanket (37°C ± 2°C). The dilator was used to separate the eyelids, and a 14 G intravenous catheter was used to insert a pressure sensor (Codman microsensor, Integra LifeSciences, NJ, USA) into the eye orbit through the location of the third eyelid. The site of the implanted pressure sensor was identified with an ultrasonic scanner (Mindray, Shenzhen, China).

### Experimental setup

**Fig 1:**
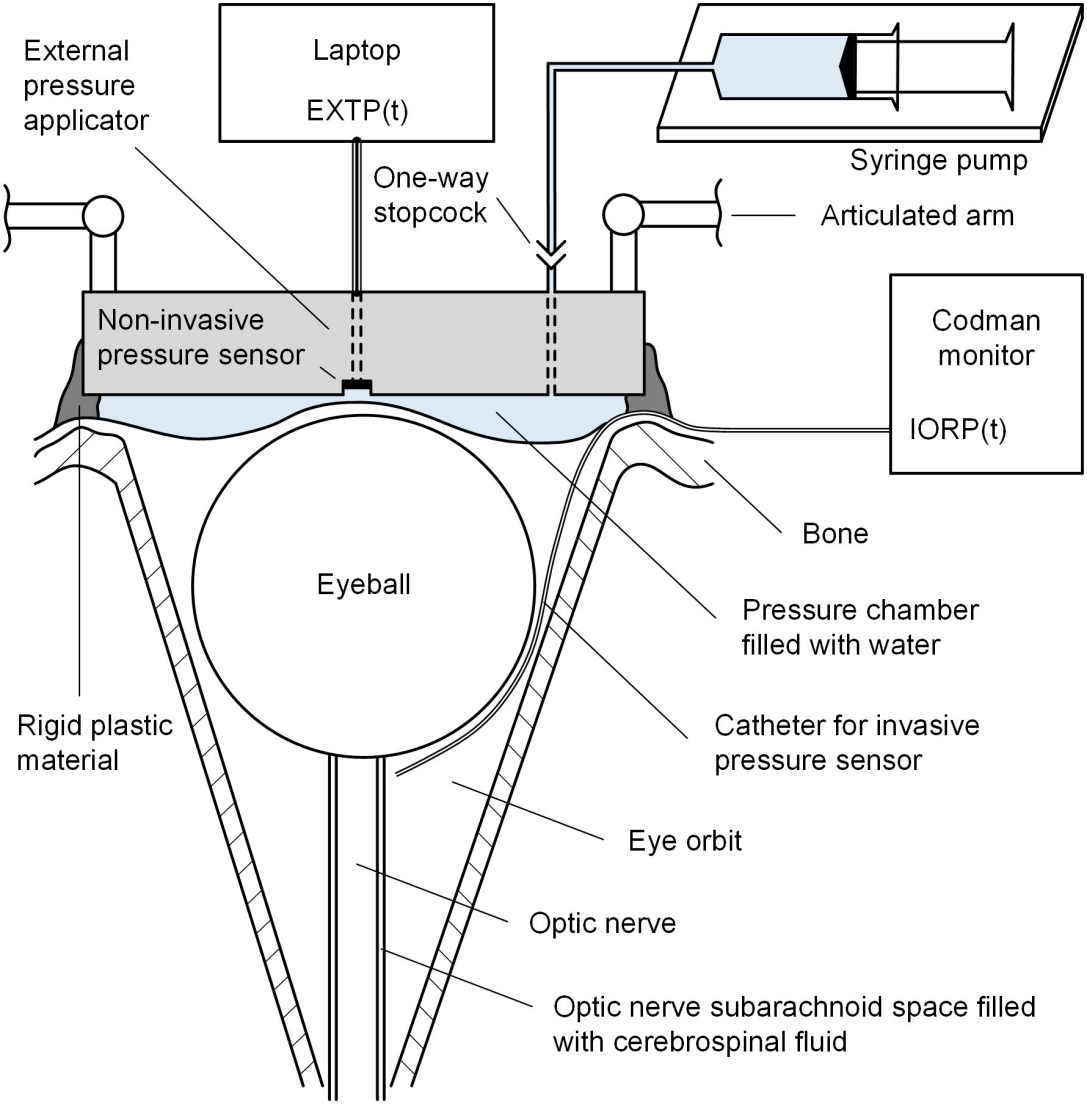
Diagram of the external pressure applicator and experimental setup. IORP(t)–intraorbital pressure over time; EXTP(t)–external pressure over time.

The experimental system consisted of a specially designed external pressure applicator that can be positioned over the orbit and tightly fixed to a bed frame using an articulated arm (Fig 1). A pressure chamber was attached at the bottom of the external pressure applicator. This chamber was made of a flexible, non-allergenic film and came into contact with the closed eyelid and the surrounding tissues of the orbit. Inside the chamber, water is injected to elevate the external pressure, thereby influencing the IORP and volume. A pressure sensor (HSCDANT001PGSA3, Honeywell, NC, USA) was installed within the external pressure applicator to obtain pressure data, which are transmitted to a laptop for display and analysis. To ensure complete hermetization between the eye socket and the external pressure applicator, a two-component plastic material, vinyl polysiloxane (Panasil putty soft, Kettenbach GmbH, Eschenburg, Germany), was applied around the external pressure applicator.

In the experimental protocol, the external pressure was incrementally raised from 0 to 20 mmHg in steps of 1 mmHg. Subsequently, it was increased from 20 to 30 mmHg in steps of 2 mmHg and then from 30 to 50 mmHg in steps of 5 mmHg. To attain each specific external pressure step in the range of 0 to 50 mmHg, the necessary volume of water was injected into the chamber. At each external pressure step, we maintained a time interval of approximately 10 to 15 seconds to record Codman readings (IORP) and the corresponding volume of injected water. This procedure was considered as the first measurement set. Next, in the second measurement set, we reversed the order of pressure steps by gradually reducing the external pressure from 50 to 0 mmHg by withdrawing water from the chamber using a syringe pump.

### Statistical analysis

The data were analyzed and processed utilizing MATLAB software (version R2021a, MathWorks). The mean values ± SD (standard deviation) of IORP, volume change within the chamber, and IORC were calculated from the measurements. The compliance *C* of certain substances within the rigid chamber with an internal volume *V* and internal pressure *P* can be determined using the following equation:

$$C=\frac{\Delta V}{\Delta P}[\frac{ml}{mmHg}]$$ (1)

where *ΔV* is the volume change, and *ΔP* is the pressure change. We conducted analyses to examine the relationships between IORP, intraorbital compliance, and the externally applied pressure on the eye socket. Additionally, we performed pressure–volume curve analyses.

## Results

We have examined 6 piglet mean age of 53.3 days, mean weight 34.5 kg. The characteristics of the individual piglet are presented in Table 1. Six measurement sets from each eye orbit were obtained from piglets 3−6. Fewer than six measurement sets per orbit were obtained for the first and second piglets due to initial technical issues. A total of 68 measurement sets were obtained from all six piglets (32 from the right orbit and 36 from the left orbit). The numbers of measurement sets obtained from individual piglets are presented in Table 2.

**Table 1:** Characteristics of the piglets included in this study.

No	Age, days	Weight, kg	Gender
1	50	29	Female
2	50	31	Female
3	70	42	Female
4	50	34	Female
5	50	36	Female
6	50	35	Female

**Table 2:** Number of measurement sets carried out in individual piglet.

No	Intraorbital pressure				Volume change			
	R orbit		L orbit		R orbit		L orbit	
	First	Second	First	Second	First	Second	First	Second
1	3	3	3	3	−	−	−	−
2	1	1	3	3	−	−	3	3
3	3	3	3	3	3	3	3	3
4	3	3	3	3	3	3	3	3
5	3	3	3	3	3	3	3	3
6	3	3	3	3	3	3	3	3
Total	16	16	18	18	12	12	15	15

R: right eye, L: left eye, First: measurement set during which external pressure was increased from 0 up to 50 mmHg according to experimental protocol, Second: measurement set during which external pressure was reduced from 50 up to 0 mmHg according to experimental protocol, Intraorbital pressure: measurement sets in which only IORP was measured, Volume change: measurement sets in which IORP and volume change inside the chamber were measured.

**Fig 2:**
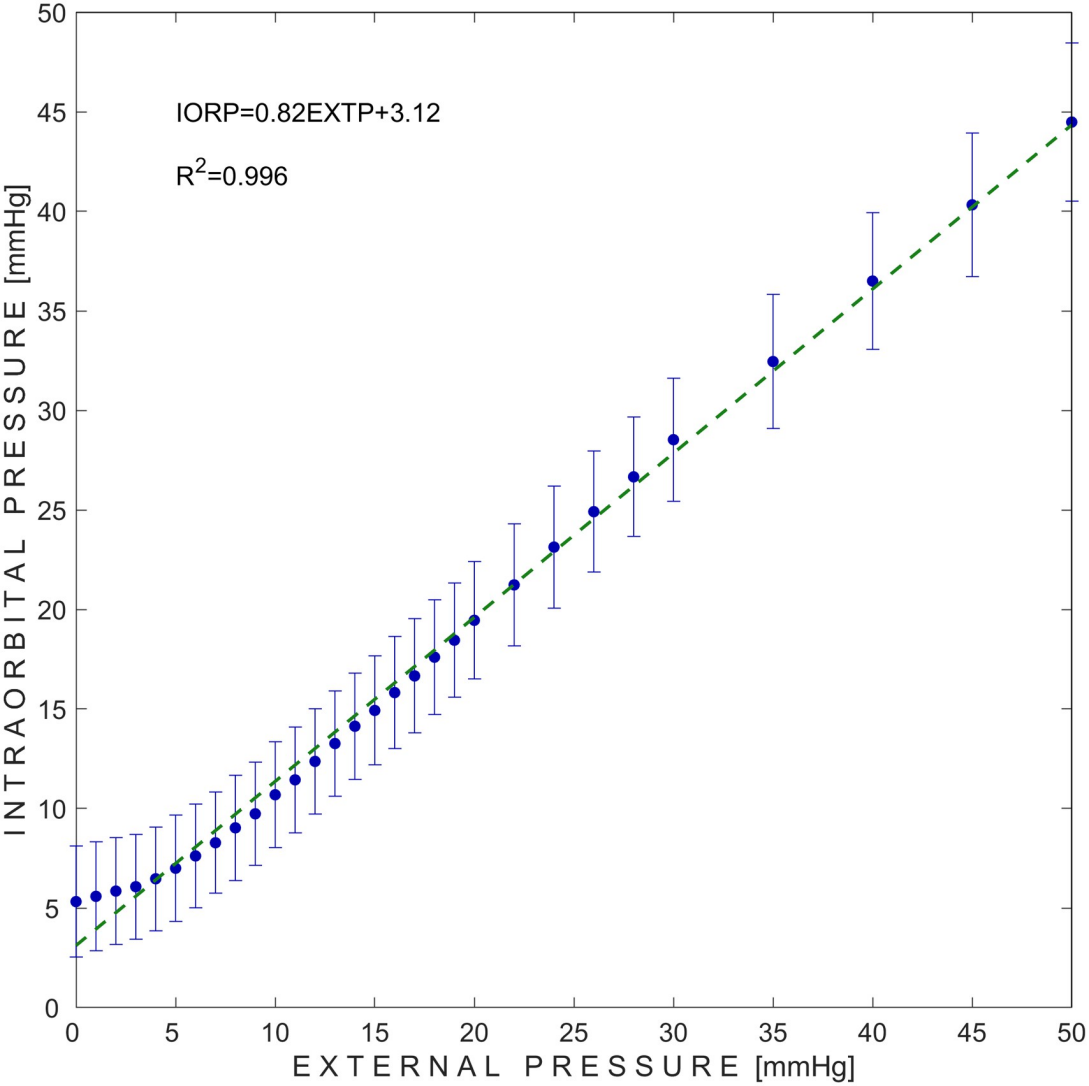
Dependence of intraorbital pressure measured using invasive Codman monitor on externally applied pressure to the orbit. Blue dots represent mean values of all 68 measurement sets, while error bars represent ±SD (standard deviation). Green dashed line represents liner approximation of the measured mean values. IORP–intraorbital pressure; EXTP–external pressure.

The dependency of IORP on externally applied pressure to the eye orbit of all 6 piglets (68 measurement sets) is presented in Fig 2. The linear approximation showed that there was a strong positive relationship (R^2^ = 0.996) between the IORP obtained with an invasive Codman monitor and the pressure applied externally to the orbit. A linear equation was obtained, IORP = 0.82EXTP+3.12, which demonstrates that external pressure can be transferred to the orbit tissues linearly with a bias (systematic error) of 3.12 mmHg which is close to initial IORP, in the tested pressure range.

**Fig 3:**
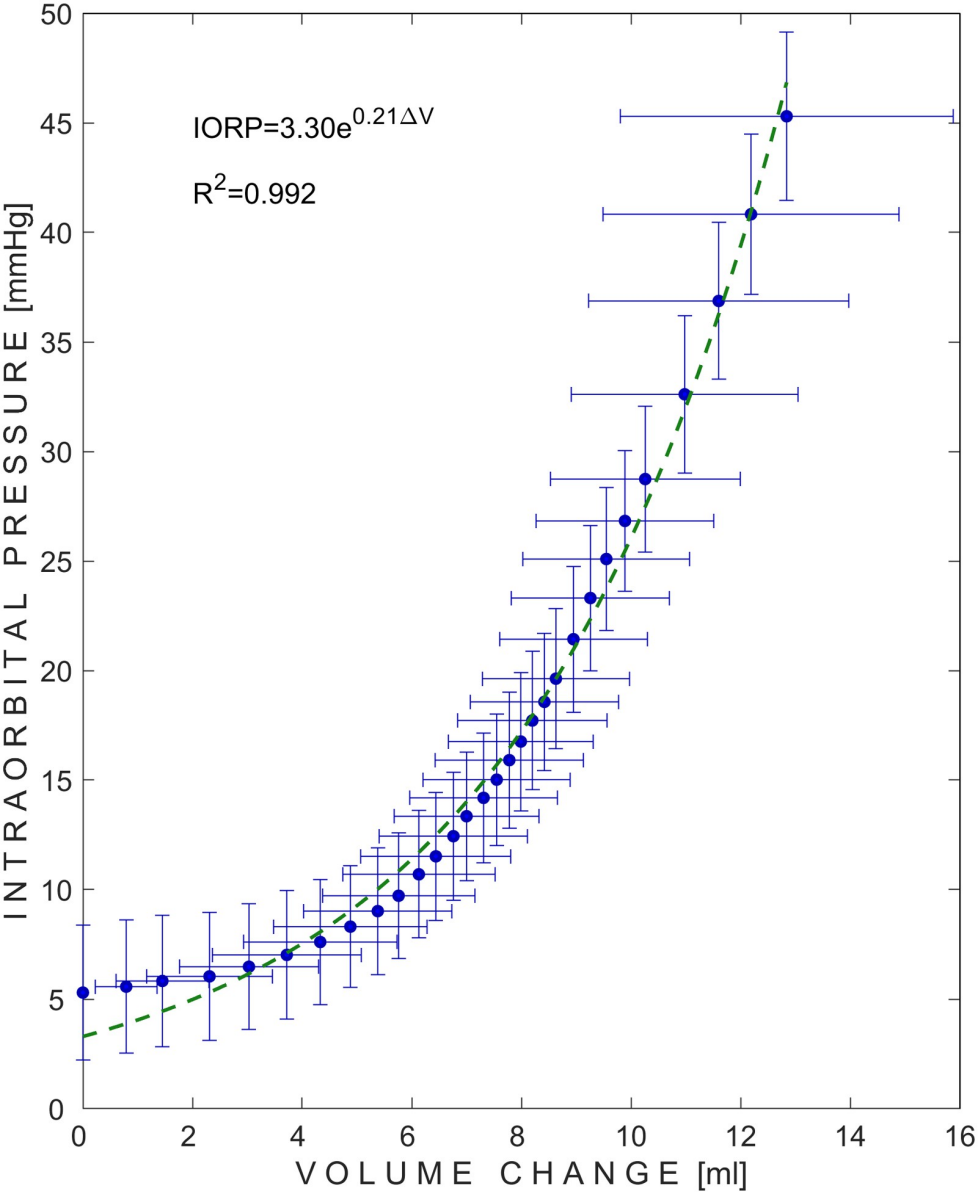
Pressure–volume curve of the orbit. Blue dots represent mean values of 54 measurement sets, while error bars represent ±SD (standard deviation). Green dashed line represents exponential approximation of the measured mean values. IORP–intraorbital pressure; ΔV–volume change.

**Fig 4:**
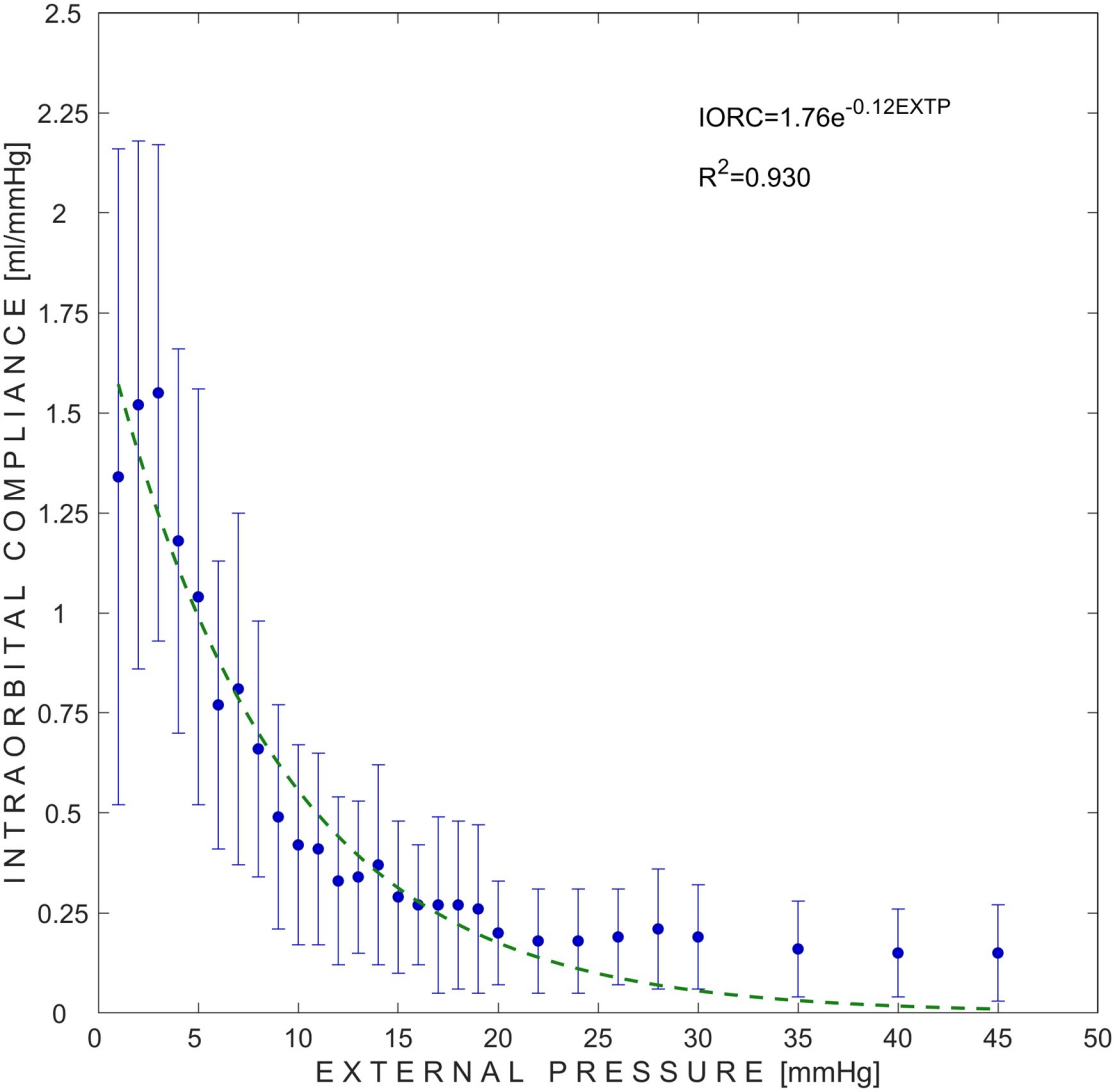
Dependense of intraorbital compliance on externally applied pressure to the orbit. Blue dots represent mean values of 54 measurement sets, while error bars represent ±SD (standard deviation). Green dashed line represents exponential approximation of the measured mean values. IORC–intraorbital compliance; EXTP–external pressure.

The dependency of IORP on the volume change of water inside the chamber of the pressure applicator from 5 piglets (54 measurement sets) is presented in Fig 3. The initial data points in Fig 3 provide a typical illustration of the venous volume being expelled from the rigid orbital cavity. Consequently, there is no rise in IORP when the volume is increased. The dependency of IORC on externally applied pressure to the orbit of all 5 piglets (54 measurement sets) is presented in Fig 4.

## Discussion

The intracranial pressure**–**volume characteristic and its nature has been described decades ago [1, 2, 13]. It is important to obtain information about the pressure**–**volume reserve capacity at which a neurosurgical or neurological patient’s brain is currently sustaied [2, 4, 14]. This can be achieved by measuring ICC together with ICP monitoring in terms of the change in volume per unit change in pressure (ΔV/ΔP) [15]. However, ICC monitoring has no standard procedure in clinical practice due to the difficulty to use, safety concerns, or lack of validation of emergent monitoring methods [11, 16].

Intraocular parameters refer to measurements taken within the eyeball, such as intraocular pressure, which should fall within the normal range of 10**–**21 mmHg for adult humans [17–19]. In contrast, intraorbital parameters refer to measurements taken within the orbit, such as IORP, which should fall within the normal range of 3**–**6 mmHg for adult humans [3, 20, 21]. In this animal study, we found an average initial IORP of 5.32 mmHg (first data point in Fig 2), while previous studies by Zoumalan et al. and Enz et al. reported mean orbital pressures of 4.1 mmHg and 2.5 mmHg, respectively [22, 23]. However, the initial data points in Fig 3 are a typical example of venous volume egress from the unyielding orbital cavity [24]. As a result, there is no elevation in IORP with an increase in volume.

The animal study showed a linear dependancy between the IORP and the pressure externally applied to the orbit (R^2^ = 0.996 in a pressure range of 0 to 50 mmHg) when it was physically confined by the pressure applicator to mimic the intracranial compartment as a rigid closed system. However, two observations should be mentioned. First, after inserting invasive pressure sensor into the orbit, the Codman device showed a non-zero values of initial pressure, even though external pressure was not applied. The invasive Codman device showed no pressure increase until the point when externally applied pressure exceeded the initial IORP. Above that value, each step increase of the external pressure was linearly transferred to the orbit. Second, the maximum externally applied pressure was set to 50 mmHg, while the average measured IORP value of all 68 measurement sets was 44.48 mmHg (SD = 3.97 mmHg). This demonstrates a clinically acceptable pressure gradient within a higher pressure range that aligns with a previously published consensus report [25].

This pilot study involved measuring the volume of water injected into a physically rigid and hermetic external pressure applicator at each externally applied pressure step in order to obtain the IORP**–**volume relationship. Exponential behavior (R^2^ = 0.992) of IORP was observed in a volume range of 0 to 12.84 ml up to a pressure of 45 mmHg, which is consistent with previous studies on humans [22, 23, 26]. Zoumalan et al. reported an increase in orbital pressure up to 68.4 mmHg after injecting 22 ml of blood into the retrobulbar space of 10 human cadaver orbits [22]. Enz et al. demonstrated an increase in IORP up to 12.8 mmHg after injecting 7 ml of 2% mepivacaine solution into the orbital compartment in 20 patients undergoing cataract surgery under local anesthesia [23].

Kim et al. measured an increase in IORP to 24.8 mmHg in response to orbital retraction up to 2.0 cm in 9 patients who underwent transorbital surgery. They also found an increase in IORP up to 35.2 mmHg in response to retraction to 2.5 cm in five cadavers [26]. Our study findings are consistent with the exponential relationship observed between intraocular pressure and volume [27, 28], as well as the intracranial pressure**–**volume relationship [29]. We have calculated the dependency of IORC on externally applied pressure and found that it can be managed within a broad range of 1.55 to 0.15 ml/mmHg. These results are in line with an earlier clinical investigation that measured ICC range between 1.407–0.141 ml/mmHg [30].

We hypothesize the potential of a non-invasive method to measure intracranial compliance by identifying the equilibrium between ICC and IORC, by a similar balancing principle [31]. This approach involves replicating the intracranial pressure–volume dynamics within the intraorbital structure and gradually introducing predetermined volumes into the pressure chamber. Nevertheless, at present, we lack any data regarding the correlation between ICC and IORC.

## Conclusions

In this pilot animal study, we observed that it is possible to transfer external pressure to the eye orbit while maintaining an acceptably low pressure gradient between intraorbital and external pressures. This was achieved by using thin elastic non-allergenic film between a rigid hermetic pressure applicator and the closed eyelid of an animal and by choosing the appropriate material for a complete hermetization between the eye socket and the applicator. It has been shown that intraorbital compliance can be controlled over a range of 1.55 to 0.15 ml/mmHg.
